# Efficacy and Safety of Vitamin K-Antagonists (VKA) for Atrial Fibrillation in Non-Dialysis Dependent Chronic Kidney Disease

**DOI:** 10.1371/journal.pone.0094420

**Published:** 2014-05-09

**Authors:** Judith Kooiman, Nienke van Rein, Bas Spaans, Koen A. J. van Beers, Jonna R. Bank, Wilke R. van de Peppel, Antonio Iglesias del Sol, Suzanne C. Cannegieter, Ton J. Rabelink, Gregory Y. H. Lip, Frederikus A. Klok, Menno V. Huisman

**Affiliations:** 1 Department of Thrombosis and Hemostasis, Leiden University Medical Center, Leiden, The Netherlands; 2 Einthoven Laboratory of Experimental Vascular Medicine, Leiden University Medical Center, Leiden, The Netherlands; 3 Department of Internal Medicine, Rijnland Hospital, Leiderdorp, The Netherlands; 4 Department of Clinical Epidemiology, Leiden University Medical Center, Leiden, The Netherlands; 5 Department of Nephrology, Leiden University Medical Center, Leiden, The Netherlands; 6 Haemostasis, Thrombosis, and Vascular Biology Unit, University of Birmingham Centre for Cardiovascular Sciences, City Hospital, Birmingham, United Kingdom; Medical University of Graz, Austria

## Abstract

**Background:**

Essential information regarding efficacy and safety of vitamin K-antagonists (VKA) treatment for atrial fibrillation (AF) in non-dialysis dependent chronic kidney disease (CKD) is still lacking in current literature. The aim of our study was to compare the risks of stroke or transient ischemic attack (TIA) and major bleeds between patients without CKD (eGFR >60 ml/min), and those with moderate (eGFR 30–60 ml/min), or severe non-dialysis dependent CKD (eGFR <30 ml/min).

**Methods:**

We included 300 patients without CKD, 294 with moderate, and 130 with severe non-dialysis dependent CKD, who were matched for age and sex. Uni- and multivariate Cox regression analyses were performed reporting hazard ratios (HRs) for the endpoint of stroke or TIA and the endpoint of major bleeds as crude values and adjusted for comorbidity and platelet-inhibitor use.

**Results:**

Overall, 6.2% (45/724, 1.7/100 patient years) of patients developed stroke or TIA and 15.6% (113/724, 4.8/100 patient years) a major bleeding event. Patients with severe CKD were at high risk of stroke or TIA and major bleeds during VKA treatment compared with those without renal impairment, HR 2.75 (95%CI 1.25–6.05) and 1.66 (95%CI 0.97–2.86), or with moderate CKD, HR 3.93(1.71–9.00) and 1.86 (95%CI 1.08–3.21), respectively. These risks were similar for patients without and with moderate CKD. Importantly, both less time spent within therapeutic range and high INR-variability were associated with increased risks of stroke or TIA and major bleeds in severe CKD patients.

**Conclusions:**

VKA treatment for AF in patients with severe CKD has a poor safety and efficacy profile, likely related to suboptimal anticoagulation control. Our study findings stress the need for better tailored individualised anticoagulant treatment approaches for patients with AF and severe CKD.

## Introduction

About one-third of atrial fibrillation (AF) patients suffer from chronic kidney disease (CKD) [1]–[3], a condition that by itself increases the risk of stroke, even in the absence of AF. Inversely, AF in CKD patients is associated with progression of CKD, cardiovascular morbidity and mortality [4]–[6].

Antithrombotic treatment is very effective in preventing stroke or a transient ischemic attack (TIA) in patients with AF, both in patients with normal renal function and in those with CKD in terms of a relative risk reduction [7]–[9]. However, CKD increases a patient's risk of major bleeding complications during antithrombotic treatment [8], [10]. The extent to which non-dialysis dependent CKD increases the risk of stroke and major bleeds in AF patients during VKA treatment is understudied, as the main focus in research in this area has been on patients with end-stage-renal disease requiring dialysis. However, these patients comprise less than 1% of the AF population [8], [11]. The few studies that have focussed on risks of stroke and/or major bleeding in AF patients with non-dialysis dependent CKD were limited by their small sample size [10], [12], [13], the absence of information on eGFR levels [8], exclusion of patients with severe CKD [7], or a divergent patient cohort with various indications for VKA treatment [14]. Knowledge about these risks would most certainly provide relevant insights into treatment outcomes in a patient group that frequently attends both cardiology and internal medicine practices. Moreover, with the emergence of novel oral anticoagulants, understanding the risks of stroke and major bleeding events in AF patients with various stages of CKD is essential when evaluating whether these new agents would provide a more favourable risk-benefit ratio than the traditional vitamin K-antagonists (VKA) for this specific patient population [11].

Therefore, the aim of our study was to compare risks of stroke or TIA and major bleeds in patients with moderate or severe CKD and AF treated with VKAs with patients without renal impairment. Second, we assessed the influence of quality of anticoagulation control on the risks of stroke or TIA and major bleeds.

## Methods

Patients diagnosed with new onset valvular or non-valvular AF starting VKA treatment between 1997 and 2005 at the Leiden anticoagulation clinic were included in a previously described study cohort [3]. This anticoagulation clinic serves one academic (Leiden University Medical Center, Leiden) and two non-academic teaching hospitals (Diaconessenhuis, Leiden, and Rijnland Hospital, Leiderdorp). Within this cohort of 5039 AF patients, 3316 had no CKD (eGFR >60 ml/min), 1557 (eGFR 30–60 ml/min) had moderate CKD, and 166 patients severe CKD (eGFR <30 ml/min), as measured at start of VKA therapy. For the current analysis, we excluded fourteen patients from the severe CKD group who had acute kidney injury at time of VKA therapy initiation, after which renal function recovered to a less critical CKD stage, thus leaving 152 patients with severe CKD. Since reviewing medical records of all 1557 moderate and 3316 non-CKD patients would be an effort not offsetting the statistical gain, we sampled 300 patients without CKD and 294 patients with moderate CKD for inclusion, matched for age and gender to those with severe CKD. Patients treated with VKA via the Leiden anticoagulation clinic for <7 days were excluded from the study cohort and replaced by others of the same age, gender, and level of renal impairment. Patients on dialysis at start of VKA therapy were also excluded, but were not replaced. Patients were treated with either phenprocoumon (Marcoumar) or acenocoumarol. The study was approved by the ethics committee of the three participating hospitals (ethics committee Leiden University Medical Center, Leiden, the Netherlands) that waived the need for informed consent.

### Chart review

Medical records from two sources (i.e. the participating hospitals and the Leiden anticoagulation clinic) were searched for information on patient characteristics at baseline, comorbidity, use of platelet-inhibitors, International Normalized Ratios (INRs) and study outcomes. Renal function was assessed using the abbreviated modification of diet in renal disease (MDRD) formula, as it accurately estimates renal function in elderly patients and the mean age of our study population was high [15], [16].

### Study outcomes and definitions

Primary outcomes of this study were the combined endpoint of stroke or TIA and the occurrence of major bleeding events. Major bleeding was defined by the International Society of Thrombosis and Hemostasis criteria (i.e. fatal bleeding, any bleeding causing a drop in hemoglobin level ≥1.24 mmol/L and/or requiring transfusion of ≥2 units of whole blood or red cells and/or a symptomatic bleeding in a critical area/organ (intracranial, intraspinal, intraocular, pericardial, or intramuscular with compartment syndrome)) [17]. Secondary endpoints were major adverse cardiovascular events (MACE), fatal MACE and fatal bleeding. MACE was defined as stroke or TIA, myocardial infarction, intermittent claudication, unstable angina, carotid endarterectomy, coronary artery bypass graft, peripheral arterial bypass or angioplasty [18]. Other variables of interest were time within therapeutic range (TTR) and INR-variability; both established risk factors for MACE and major bleeding complications during VKA treatment [19]–[23]. INR-values were measured using HepatoQuick (Roche Diagnostics, Mannheim, Germany) and serum creatinine values by using Roche Diagnostics Analyzers (Mannheim, Germany).

### Follow- up

Duration of follow-up was defined as time elapsed between the day of initiation and permanent discontinuation of VKA treatment, occurrence of the endpoint of interest, or death, or December 31, 2010. Only the first stroke or TIA, MACE or major bleeding event that occurred was recorded in the database, although some patients had more than one episode of the endpoints. For non-acute MACE such as intermittent claudication, carotid endarterectomy, coronary artery bypass graft, peripheral arterial bypass or angioplasty, the day of diagnosis or intervention was recorded as the date of MACE occurrence.

### Statistical analysis

Incidence rates (i.e. events/100 patient years (py)) of primary outcomes were reported with corresponding 95% confidence intervals (CI). As patients with moderate or without CKD were matched for age and gender to those with severe CKD, incidence rates of our study endpoints in the first two patient groups reflect the incidences of these endpoints for the population with this age and sex distribution, rather than that of the overall AF population. Nonetheless, this design allowed us to study relative risks of stroke or TIA and major bleeds between patients with severe or moderate CKD and those without renal impairment, which was our main research question. This analysis was performed using uni- and multivariate Cox regression analyses reporting hazard ratios (HRs) as crude values and as values corrected for age, gender, concomitant use of platelet-inhibitors or non-steroidal anti-inflammatory drugs, hypertension, diabetes mellitus, and congestive heart failure. As for secondary analysis, TTR and INR-variability were calculated using the *Rosendaal* method and the formula by *Cannegieter*, respectively [23], [24]. INR-variability and TTR were compared between patients with moderate or severe CKD and non-CKD patients, using first a Kruskal Wallis and second a Mann Whitney U-test, as these values had a non-parametric distribution among the population.

### Mediation analysis

A mediation analysis was performed to assess whether the expected increased risks of stroke or TIA, major bleeds and MACE in patients with moderate and severe CKD compared with non-CKD patients were mediated via TTR or INR-variability. This analysis was performed for the three endpoints and the combined endpoint of MACE and major bleeds in a nested case-control study [24]. We chose this design as the duration of follow-up needed to be matched for cases and controls. Cases were patients developing the endpoint of interest. For each case, a maximum of four controls was selected from the total study population of 724 patients who were treated during the same period with VKA while not developing this specific endpoint at the time that the case did (incidence density sampling). A control could be selected for more than one case. INR-variability and TTR were calculated over the entire treatment period, for the last six and the last three months prior to the outcome of interest. For each time frame, only patients with sufficient follow-up were selected for that specific analysis [24]. Crude odds ratios (OR) were then computed for the risks of the four outcomes comparing severe and moderate CKD with non-CKD patients. Next, these ORs were first adjusted for comorbidity, and second for either INR-variability, TTR, or both for each individual time frame.

All statistical analyses were performed in SPSS 20.0 (IBM SPSS statistics, IBM Corp, Somers, NY).

## Results

**Figure 1:**
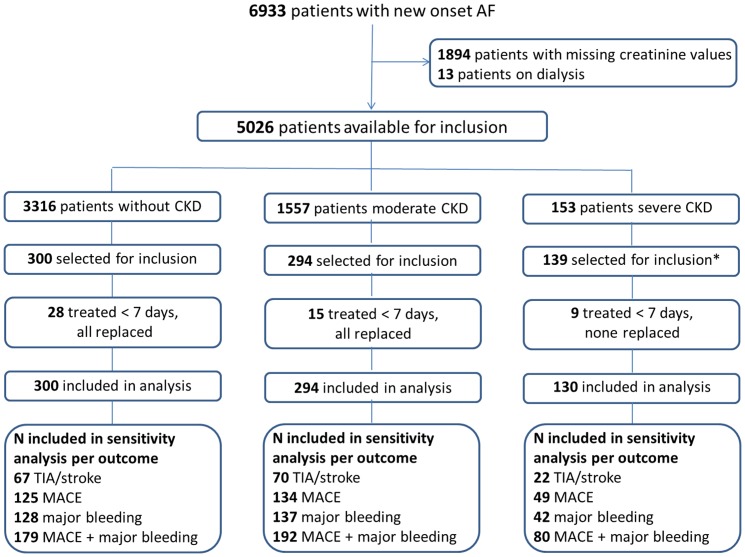
Flow chart. Abbreviations: CKD  =  Chronic kidney disease, TIA  =  transient ischemic attack, MACE  =  major adverse cardiovascular event. * Fourteen patients from the severe CKD group had acute kidney injury at time of VKA therapy initiation, after which renal function recovered to a less critical CKD stage.

Serum creatinine values were available in 5039 out of 6933 patients with new onset AF at start of VKA therapy. Of those, 733 matched subjects were selected for inclusion for this present study, comprising all patients with non-dialysis depended severe CKD, and a sample of those with moderate or without CKD. Registered duration of VKA treatment in the Leiden anticoagulation clinic was less than seven days in 52 patients who were excluded and replaced by 43 patients of similar gender, age, and level of renal impairment. The remaining nine severe CKD patients could not be replaced (Figure 1). Thus, 724 patients were included in this study, 300 without CKD (eGFR >60 ml/min), 294 with moderate (eGFR 30–60 ml/min) and 130 with severe CKD (eGFR <30 ml/min). Patient characteristics at baseline are reported in Table 1. Compared with patients without CKD, those with moderate or severe CKD were more likely to have congestive heart failure, hypertension, diabetes mellitus, or a previous episode of major bleeds before initiation of VKA therapy. Median follow-up time was 2.1 years (2.5–97.5percentile 0.0–10.0) for the endpoint of stroke or TIA and 2.3 years (2.5–97.5percentiles 0.0–10.0) for major bleeding events.

**Table 1:** Patient characteristics at baseline of the total population.

		No CKD eGFR>60 ml/min	Moderate CKD eGFR 30–60 ml/min	Severe CKD eGFR<30 ml/min
N		300	294	130
Mean age (SD)		74(10)	75(10)	76(9)
Gender, m/f		171/129	165/129	73/57
Mean eGFR (SD)		92(37)	46(8)	21(7)
Diabetes Mellitus		35(11.7)	50(17.0)	38(29.2)
Hypertension		138(46.0)	181(61.6)	80(61.5)
Concomitant use of platelet inhibitors		26(8.7)	35(11.9)	7(5.4)
Previous stroke or TIA		47(15.7)	59(20.1)	21(16.2)
Previous major bleeding		14(4.7)	21(7.1)	10(7.7)
Congestive heart failure		50(17.0)	105(35.7)	54(41.5)
INR target range*				
	2.0–3.0	1(0.3)	0(0.0)	1(0.8)
	2.5–3.5	287(98.6)	280(97.9)	116(97.5)
	3.0–4.0	3(1.0)	6(2.1)	2(1.7)
Acenocoumarol**		13(4.3)	15(5.1)	9(7.0)
Phenprocoumon		287(95.7)	278(94.6)	120(93.0)

Data is presented as n, % unless stated otherwise.

CKD  =  chronic kidney disease, eGFR  =  estimated glomerular filtration rate, SD  =  standard deviation, TIA  =  transient ischemic attack.

* Lacking in 30 patients, ** lacking in one patient.

### Stroke or TIA and MACE

During follow-up for the primary endpoint, 6.2% (45/724, 1.67/100 py) of patients developed a stroke (29 patients) or TIA (16 patients). The risk of stroke or TIA was increased in those with severe CKD compared with patients without renal dysfunction (HR 2.75, 95%CI 1.25–6.05) or those with moderate CKD (HR 3.93, 95%CI 1.71–9.00, Table 2). The risk of stroke or TIA was similar for patients with moderate and without CKD.

**Table 2:** Risk of stroke, TIA, MACE and major bleeding events during vitamin K-antagonist treatment stratified by renal function within the entire population.

Endpoint	No. of events	N/100 py‡ (95% CI)	Crude HR (95% CI)	Adjusted HR (95% CI)
**Stroke or TIA**				
No CKD	19	1.62(1.03–2.54)	ref	ref
Moderate CKD	15	1.20(0.71–1.99)	0.72(0.36–1.41)	0.70(0.35–1.41)
Severe CKD	11	4.24(2.30–7.53)	2.40(1.13–5.07)	2.75(1.25–6.05)
**Overall MACE** *				
No CKD	36	3.48(2.51–4.80)	ref	ref
Moderate CKD	41	3.79(2.80–5.12)	1.06(0.68–1.67)	1.05(0.66–1.67)
Severe CKD	30	15.4(10.95–21.16)	3.78(2.31–6.19)	3.57(2.10–6.06)
**Fatal MACE** †				
No CKD	7	0.64(0.28–1.35)	ref	ref
Moderate CKD	12	1.05(0.58–1.85)	1.68(0.66–4.27)	1.64(0.64–4.17)
Severe CKD	4	1.67(0.50–4.38)	2.09(0.60–7.23)	1.92(0.55–6.67)
**Overall major bleeding** *				
No CKD	46	4.45(3.34–5.89)	ref	ref
Moderate CKD	45	4.11(3.07–5.46)	0.91(0.60–1.37)	0.90(0.59–1.37)
Severe CKD	22	8.84(5.85–13.07)	1.88(1.13–3.14)	1.66(0.97–2.86)
**Fatal bleeding** †				
No CKD	9	0.83(0.41–1.59)	ref	ref
Moderate CKD	8	0.70(0.33–1.40)	0.85(0.33–2.21)	0.82(0.32–2.13)
Severe CKD	4	1.67(0.50–4.38)	1.62(0.49–5.33)	1.52(0.46–5.02)

Definitions: no-CKD  =  estimated glomerular filtration rate (eGFR) >60 ml/min, moderate CKD  =  eGFR 30–60 ml/min, severe CKD  =  eGFR <30 ml/min.

Abbreviations: CKD  =  chronic kidney disease, PY  =  patient years, CI  =  confidence interval, HR  =  hazard ratio, MACE  =  major adverse cardiovascular event, TIA  =  transient ischemic attack.

‡ Reported incidences for patients with an eGFR 30–60 or eGFR >60 ml/min are influenced by sampling of patients matched for age and gender to those with an eGFR <30 ml/min.

* HR adjusted for age, gender, hypertension, the use of platelet-inhibitors, diabetes mellitus and congestive heart failure.

† HR adjusted for age and gender. Further correcting resulted in non-converging coefficients.

Overall, 14.8% (107/724) of patients developed MACE of whom 28 patients had a stroke, 15 a TIA, 28 a myocardial infarction, 17 an unstable angina pectoris, 11 patients underwent coronary artery bypass grafting, and 8 patients had developed peripheral artery disease (one patient developed a stroke and another patient a TIA after the occurrence of an earlier MACE). Patients with severe CKD were at increased risk of MACE compared with non-CKD patients (adjusted HR 3.57, 95%CI 2.10–6.06) and those with moderate CKD (adjusted HR 3.40, 95%CI 2.05–5.64). MACE risk was similar for those without and with moderate CKD. Twenty-three of 724 patients had a fatal MACE, of whom 14 (60.9%) developed a myocardial infarction and 9 (39.1%) a stroke. Although non-significant, moderate and severe CKD were associated with a 60–90% increased risk of fatal MACE compared with non-CKD patients, respectively.

### Major bleeding complications

Major bleeding complications occurred in 15.6% of patients (113/724, 4.8/100 py). Although non-significant, severe CKD was associated with an increased risk of major bleeds compared with patients without renal impairment (adjusted HR 1.66, 95%CI0.97–2.86), and those with moderate CKD (HR 1.86, 95%CI1.08–3.21). Major bleeding risks were similar for those without and with moderate CKD. The most frequent locations of major bleeding were gastrointestinal (34.5%) and intracranial (27.4%) in the total population. Patients with severe CKD were more likely to develop gastrointestinal bleeding (63.6%), yet less frequently developed intracranial haemorrhages (13.6%).

Fatal bleeding occurred in 2.9% of patients (21/724). Severe CKD might be associated with an increased risk of fatal bleeding events compared with non-CKD patients (HR 1.52, 95%CI0.46–5.02, Table 2), and those with moderate CKD (HR 1.90, 95%CI0.57–6.41).

### TTR and INR variability

Compared with patients without CKD, TTR was higher in those with moderate CKD (75.1%, p<0.01) whereas TTR was similar in patients with severe CKD (70.3%, p = 0.41, Table 3). The proportion of time spent above target range was higher for all CKD stages compared with patients without renal impairment, and higher for those with severe compared with moderate CKD. Median INR-variability during the entire treatment period significantly increased with each stage of CKD, with median values of 0.5 in patients without CKD, 0.7 (p = 0.03) in those with moderate, and 0.9 (p<0.001) in those with severe CKD. For all three groups, the degree of INR variability can be regarded as below average or unstable anticoagulant control according to previous research [25].

**Table 3:** Time within therapeutic range and INR variability within the entire population of 724 patients with atrial fibrillation.

		No CKD	Moderate CKD	P-value comparison	Severe CKD	P-value comparison	P-value comparison with
		eGFR>60 ml/min	eGFR 30-60 ml/min	with no CKD patients	eGFR<30 ml/min	with no CKD patients	moderate CKD patients
**Time spend within therapeutic range, %**							
First six weeks of VKA therapy		39.4(13.2–73.5)	49.7(24.1–81.3)	0.01	44.1(26.4–77.9)	0.10	0.60
First eighteen weeks of VKA therapy		57.9(29.8–79.3)	65.5(42.1–83.9)	0.01	60.7(39.4–80.6)	0.37	0.19
First twenty-six weeks of VKA therapy		61.5(38.7–79.8)	67.1(46.7–82.4)	0.02	64.7(41.5–75.6)	0.92	0.07
Entire treatment period		67.0(43.1–81.1)	75.1(57.8–82.9)	<0.01	70.3(49.2–81.1)	0.41	0.10
**Time under target range (entire treatment), %**		8.7(2.6–35.5)	6.2(2.1–13.0)	<0.001	5.5(2.3–12.9)	0.001	0.77
**Time above target range (entire treatment), %**		11.7(3.9–21.2)	15.2(9.8–24.0)	<0.001	20.8(11.7–32.7)	<0.001	<0.01
**INR variability (2.5-97.5 percentiles)**							
First six weeks of VKA therapy		0.5(0.1–1.6)	0.6(0.2–1.6)	0.10	0.7(0.4–2.3)	0.001	0.03
First eighteen weeks of VKA therapy		0.4(0.2–1.3)	0.6(0.2–1.5)	0.08	0.8(0.4–1.8)	<0.001	0.01
First twenty-six weeks of VKA therapy		0.5(0.3–1.2)	0.7(0.4–1.2)	0.24	0.8(0.4–1.8)	<0.001	<0.01
Entire treatment period		0.5(0.3–1.2)	0.7 (0.4–1.2)	0.03	0.9(0.5–1.8)	<0.001	<0.01

Data are presented as median, (Interquartile range), P-values were computed using Mann-Whitney test, after proof of significant differences between groups using a Kruskal-Wallis test.

CKD  =  chronic kidney disease, VKA  =  vitamin K-antagonists, eGFR  =  estimated glomerular filtration rate, INR  =  international normalized ratio.

### Mediation analysis

Mediation analyses were performed on the influence of TTR and INR-variability on the increased risks of stroke or TIA, MACE and major bleeding complications in severe CKD compared with non-CKD patients. TTR and INR-variability were analysed as continuous and categorical variables (based on 33^rd^ and 66^th^ percentiles) in separate models demonstrating similar results. For all four outcomes, the results demonstrated a decrease in the odds ratio towards unity in severe CKD compared with non-CKD patients, when corrected for either INR-variability or TTR (Table 4 and 5). However, the effect of INR-variability and TTR in the three months prior to combined endpoint of stroke or TIA and to the endpoint of MACE was less pronounced. This might be the result of the low number of INR measurements during this short timeframe (median 5.0, 2.5–97.5 percentile 2.0–10.5), which might not be sufficient for adequate assessment of TTR and INR-variability. Simultaneous correction for both INR-variability and TTR did not result in a further decrease towards unity for any of the endpoints comparing severe and moderate CKD to non-CKD patients, indicating no additive effect of TTR over INR-variability, and vice versa. This indicates that the increased risks in patients with severe CKD for stroke or TIA, major bleeds and MACE were mediated via suboptimal anticoagulation control.

**Table 4:** Mediation analysis on effect of INR-variability on the increased risks of major adverse cardiovascular events and major bleeding complications in patients with chronic kidney disease in a nested case-control study.

Outcome	Crude OR (95% CI)	Adjusted OR 1 (95% CI)	Adjusted OR 2 (95% CI)	Adjusted OR 3 (95% CI)	Adjusted OR 4 (95% CI)
**Stroke or TIA**					
No CKD (N = 91, of whom 67 unique)	ref	ref	ref	ref	ref
Moderate CKD (N = 90, of whom 70 unique)	0.76(0.36–1.61)	0.76(0.35–1.67)	0.79(0.36–1.76)	1.06(0.41–2.75)	1.02(0.40–2.56)
Severe CKD (N = 25, of whom 22 unique)	2.98(1.17–7.60)	2.56(0.92–7.10)	2.23(0.73–6.84)	1.96(0.50–7.74)	2.50(0.69–9.07)
**MACE**					
No CKD (N = 210, of whom 125 unique)	ref	ref	ref	ref	ref
Moderate CKD (N = 205, of whom 134 unique)	1.21(0.74–1.98)	1.14(0.68–1.93)	1.15(0.66–1.99)	1.05(0.58–1.91)	1.10(0.62–1.96)
Severe CKD (N = 58, of whom 49 unique)	5.18(2.76–9.70)	5.07(2.57–10.02)	5.37(2.58–11.19)	3.58(1.59–8.03)	3.77(1.71–8.32)
**Major bleeding**					
No CKD (N = 211, of whom 128 unique)	ref	ref	ref	ref	ref
Moderate CKD (N = 245, of whom 137 unique)	0.81(0.51–1.28)	0.76(0.47–1.23)	0.74(0.45–1.22)	0.88(0.52–1.52)	0.83(0.49–1.40)
Severe CKD (N = 60, of whom 42 unique)	2.08(1.12–3.85)	1.77(0.91–3.43)	1.82(0.93–3.56)	1.55(0.70–3.44)	1.57(0.72–3.40)
**Major bleeding or MACE**					
No CKD (N = 361, of whom 179 unique)	ref	ref	ref	ref	ref
Moderate CKD (N = 419, of whom 192 unique)	0.87(0.62–1.25)	0.85(0.59–1.24)	0.87(0.59–1.27)	0.93(0.61–1.41)	0.88(0.59–1.34)
Severe CKD (N = 122, of whom 80 unique)	2.92(1.88–4.54)	2.61(1.64–4.16)	2.67(1.64–4.34)	2.49(1.40–4.41)	2.31(1.33–4.03)

Model 1 includes age, gender, hypertension, the use of platelet-inhibitors, diabetes mellitus and congestive heart failure.

Model 2 includes model 1 + INR VAR entire treatment period, Model 3 includes model 1 + INR variability over six months prior to event.

Model 4 includes model 1 + INR variability over three months prior to event.

Abbreviations: CKD  =  chronic kidney disease, MACE  =  major adverse cardiovascular event, OR  =  odds ratio, TIA  =  transient ischemic attack.

**Table 5:** Mediation analysis on effect of time within therapeutic range on the increased risks of major adverse cardiovascular events and major bleeding complications in patients with chronic kidney disease in a nested case-control study.

Outcome	Crude OR (95% CI)	Adjusted OR 1 (95% CI)	Adjusted OR 2 (95% CI)	Adjusted OR 3 (95% CI)	Adjusted OR 4 (95% CI)
**Stroke or TIA**					
No CKD (N = 91, of whom 67 unique)	ref	ref	ref	ref	ref
Moderate CKD (N = 90, of whom 70 unique)	0.76(0.36–1.61)	0.76(0.35–1.67)	0.94(0.41–2.13)	1.31(0.52–3.37)	1.01(0.40–2.56)
Severe CKD (N = 25, of whom 22 unique)	2.98(1.17–7.60)	2.56(0.92–7.10)	1.95(0.64–5.88)	2.49(0.65–9.55)	2.46(0.68–8.88)
**MACE**					
No CKD (N = 210, of whom 125 unique)	ref	ref	ref	ref	ref
Moderate CKD (N = 205, of whom 134 unique)	1.21(0.74–1.98)	1.14(0.68–1.93)	1.16(0.67–2.01)	1.06(0.58–1.93)	1.11(0.62–1.98)
Severe CKD (N = 58, of whom 49 unique)	5.18(2.76–9.70)	5.07(2.57–10.02)	4.98(2.42–10.23)	3.26(1.45–7.34)	3.42(1.55–7.55)
**Major bleeding**					
No CKD (N = 211, of whom 128 unique)	ref	ref	ref	ref	ref
Moderate CKD (N = 245, of whom 137 unique)	0.81(0.51–1.28)	0.76(0.47–1.23)	0.73(0.44–1.21)	0.87(0.51–1.50)	0.83(0.49–1.41)
Severe CKD (N = 60, of whom 42 unique)	2.08(1.12–3.85)	1.77(0.91–3.43)	1.55(0.79–3.07)	1.48(0.67–3.29)	1.50(0.68–3.28)
**Major bleeding or MACE**					
No CKD (N = 361, of whom 179 unique)	ref	ref	ref	ref	ref
Moderate CKD (N = 419, of whom 192 unique)	0.87(0.62–1.25)	0.85(0.59–1.24)	0.93(0.62–1.40)	0.96(0.63–1.46)	0.93(0.62–1.40)
Severe CKD (N = 122, of whom 80 unique)	2.92(1.88–4.54)	2.61(1.64–4.16)	2.22(1.27–3.88)	2.37(1.33–4.21)	2.22(1.27–3.88)

Model 1 includes age, gender, hypertension, the use of platelet-inhibitors, diabetes mellitus and congestive heart failure.

Model 2 includes model 1 + time within therapeutic range over entire treatment period, Model 3 includes model 1 + time within therapeutic range over six months prior to event, Model 4 includes model 1 + time within therapeutic range over three months prior to event.

Abbreviations: CKD  =  chronic kidney disease, MACE  =  major adverse cardiovascular event, OR  =  odds ratio, TIA  =  transient ischemic attack.

## Discussion

Our study has three important findings. First, risks of stroke or TIA, MACE and major bleeding complications during VKA therapy were high in AF patients with severe non-dialysis dependent CKD, when compared to those without renal impairment, or with moderate CKD. Second, stroke or TIA, MACE and major bleeding risks were similar for patients with moderate CKD and those with normal renal function. Third, patients with CKD spent more time above INR target range and had a higher INR-variability. Consequently, in a nested case-control study we have shown additionally that poor anticoagulation control was associated with increased risks of stroke or TIA, MACE and major bleeds in severe CKD patients. Our study therefore provides important insights into the efficacy and safety of VKA treatment in patients with CKD and AF.

CKD is a common comorbid condition in AF patients and increases a patient's risk for both stroke and major bleeds. Suggested mechanisms for this higher stroke and bleeding risk are endothelial dysfunction, hypercoagulability, and chronic inflammation [8], [11], [12]. We demonstrated in a nested case-control study that impaired anticoagulation control might be an important additional determinant. Interestingly, within the total study population of 724 patients, CKD patients were spending more time above INR target range and had a higher INR variability compared with non-CKD patients, despite frequent INR monitoring. We hypothesize several explanations for this observation. CKD by itself may affect the quality of anticoagulant treatment. First, renal impairment might influence hepatic VKA metabolism, as has been shown in animal models for hepatic cytochrome P-450 metabolism [26], [27]. Second, CKD influences the pharmacokinetic characteristics of VKA, as warfarin half-life was reported to be shorter in CKD compared with non-CKD patients with a greater unbound warfarin fraction [28], [29]. Third, we cannot exclude that anticoagulant control is impaired in patients with CKD by poor patient compliance. Regardless of the mechanism by which CKD influences the quality of VKA therapy, our nested case-control study indicates that the increased risks of stroke or TIA, MACE and major bleeding complications in severe CKD patients are mediated through suboptimal anticoagulation control. This suggests that although warfarin has been shown to be effective in preventing stroke in CKD patients with AF in two observational and one randomized study [7], [8], [12], there is a great need for better tailored anticoagulant treatment approaches for this specific population, involving either better INR control, or the use of anticoagulants other than VKAs.

The use of computer-assisted dosage programs surveying both INR-variability and TTR during VKA treatment may help to identify patients with poor anticoagulant control in order to prevent them from developing stroke, TIA or major bleeding events [30]. Further, patient education and self-monitoring of INRs might improve patient compliance [31].

The novel oral anticoagulants have demonstrated less inter- and intra-individual variability in their pharmacokinetic properties compared with VKA. Within the Phase-3 trials, subgroup analyses have been performed for the efficacy and safety of these new agents compared with standard warfarin or aspirin treatment in AF patients with moderate CKD (i.e. eGFR >25 or >30 ml/min) [32]. These analyses demonstrated either a reduced risk of stroke and systemic thromboembolism compared with warfarin (Dabigatran 150 mg twice daily) or aspirin (Apixaban 5 mg twice daily), or a similar efficacy compared with warfarin treatment for AF (rivaroxaban 20 mg per day, or apixaban 5 mg twice daily). In terms of safety, the Aristotle trial demonstrated a lower risk of major bleeds in the apixaban compared with the warfarin group, whereas for all other novel oral anticoagulants, bleeding risks were comparable with the risks on warfarin or aspirin treatment [32]. Though, it is unknown whether these new agents would provide a better tailored anticoagulant treatment strategy compared with warfarin in severe CKD patients as they have been excluded systematically from the Phase-3 trials [33]–[35]. In terms of renal clearance, apixaban (25%) and betrixaban (17%) might be most suitable for use in patients with CKD. Although betrixaban has not been studied yet in a Phase-3 trial for stroke prevention in AF, the results of the Phase-2 trial, in which only CKD patients on dialysis were excluded, were promising in terms of a lower risk of bleeding compared with standard warfarin treatment in the group receiving the lowest betrixaban dose (i.e. 40 mg daily) [36]. However, the use of novel oral anticoagulants may not be advisable when the insufficient anticoagulant control in the CKD population is caused by poor patient compliance, which might even more be difficult to manage when laboratory monitoring of anticoagulant therapy is no longer required.

Our study has limitations. First, we had no information on alterations in renal function during follow-up, which might have led to misclassification of patients and consequently a misestimation of the reported hazard ratios. Second, events were recorded from chart review given the design of the study and we cannot fully exclude that some events were missed. However, our endpoints of stroke or TIA, MACE and major bleeding were clearly defined and are both serious medical events, requiring evaluation in a hospital setting and are thus likely to be reported in medical charts. Third, our primary and secondary outcomes were not adjudicated by an independent committee. Fourth, our sample size was too small to make further subdivisions in the stages of CKD other than moderate and severe CKD, or to demonstrate statistical differences in fatal MACE and bleeding rates. Fifth, we did not investigate the influence of co-medication interacting with VKAs on study outcomes. However, the majority of medication used by severe CKD patients is not known for interactions with VKAs. Sixth, we missed serum creatinine values at time of VKA initiation in 1894 of 9633 patients (19.6%) but we selected all patients with severe CKD (for whom serum creatinine values are highly unlikely to be lacking) and sampled controls matched for age and gender with moderate or without CKD. As patients without serum creatinine values are unlikely to have severe CKD it is implausible that lacking creatinine values in 1894 patients influenced the reported HRs on study outcomes.

In conclusion, patients with severe non-dialysis dependent CKD (i.e. eGFR <30 ml/min) are at higher risk for stroke or TIA, MACE and major bleeding complications during VKA treatment for AF, compared with those with moderate CKD (i.e. eGFR 30–60), or without renal impairment. Our study suggests that suboptimal anticoagulation control is a determinant in their poor cardiovascular prognosis. These study findings stress the need for more advanced tailored anticoagulant treatment approaches for AF patients with severe CKD. Whether the use of computer-assisted VKA dosage programs monitoring both INR-variability and TTR, or the use of novel oral anticoagulants are the answer to this issue remains to be studied.
